# Bilateral vestibulopathy: a clinical update and proposed diagnostic algorithm

**DOI:** 10.3389/fneur.2023.1308485

**Published:** 2023-12-19

**Authors:** Lisa van Stiphout, David J. Szmulewicz, Nils Guinand, Angélica Pérez Fornos, Vincent Van Rompaey, Raymond van de Berg

**Affiliations:** ^1^Department of Otorhinolaryngology and Head and Neck Surgery, Division of Balance Disorders, School for Mental Health and Neuroscience, Maastricht University Medical Center, Maastricht, Netherlands; ^2^Royal Victorian Eye and Ear Hospital, University of Melbourne, Melbourne, VIC, Australia; ^3^Bionics Institute, Melbourne, VIC, Australia; ^4^Service of Otorhinolaryngology Head and Neck Surgery, Department of Clinical Neurosciences, Geneva University Hospitals, Geneva, Switzerland; ^5^Department of Otorhinolaryngology, Head and Neck Surgery, Antwerp University Hospital, Antwerp, Belgium; ^6^Faculty of Medicine and Health Sciences, University of Antwerp, Antwerp, Belgium

**Keywords:** bilateral vestibulopathy, clinical update, diagnostic algorithm, diagnosis, review, vestibular impairment, vestibulopathy

## Abstract

Bilateral vestibulopathy (BVP) is characterized by its heterogeneous and chronic nature with various clinical presentations and multiple etiologies. This current narrative review reflects on the main insights and developments regarding clinical presentation. In addition, it proposes a new diagnostic algorithm, and describes available and potential future therapeutic modalities.

## 1 Background

Bilateral vestibulopathy (BVP) was first described in 1936 in patients with Menière’s disease who had been managed with bilateral vestibular neurectomy (1, 2). BVP has also been known as Dandy syndrome (after the neurosurgeon who performed 907 vestibular neurectomies), bilateral vestibular hypofunction, bilateral vestibular impairment, bilateral vestibular areflexia and bilateral vestibular loss (3–5). The Consensus document of the Classification Committee of the Bárány Society (2017) recommends “bilateral vestibulopathy” as the preferred term (6). As the variation in the terms for BVP imply, it is defined by a bilaterally reduced or absent function of the vestibular end organs and/or nerves, ganglia, the vestibular root entry zone and/or the brain, which negatively impacts vestibular functioning resulting in symptoms of impaired gaze stabilization and imbalance (7). The reported prevalence varies from 28 to 81 per 100,000 people. However, this is believed to be a significant under estimation based on misdiagnosis (8–12). This is partly caused by the heterogeneous presentation of the disorder, with its various clinical characteristics and multiple etiologies (5, 7, 13, 14). BVP negatively impacts quality of life and the socio-economic burden of BVP is substantial, due to work-related disability and health service utilization (8, 15–17). Here we offer an evidence-based approach for the clinician in approaching the patient with a potential BVP.

## 2 Clinical characteristics

### 2.1 Etiology

BVP may be the result of over 20 different etiologies (Table 1) (7). Nonetheless, the reported percentages of idiopathic BVP vary between 20 and 75% (7, 14, 19–21). The more common causes of BVP are genetic disorders (e.g., DFNA9), ototoxicity exposure (e.g., aminoglycosides antibiotics, chemotherapy), and infectious causes (e.g., meningitis). Less frequently, BVP may be caused by bilateral Menière’s Disease, trauma, auto-immune disease [e.g., Cogan’s syndrome, Autoimmune Inner Ear Disease (AIED)], and neurodegenerative disorders [e.g., Cerebellar Ataxia with Neuronopathy and Vestibular Areflexia Syndrome (CANVAS)] (19, 22, 23). BVP may also be a component of peripheral neuropathy [e.g., Chronic Inflammatory Demyelinating Polyradiculoneuropathy (CIDP) and Charcot–Marie Tooth (CMT) disease], congenital syndromes (e.g., Usher and Turner syndromes) and Wernicke’s encephalopathy (19, 24). Furthermore, an association between vestibular migraine and the development of BVP has been described (7, 25). Largely depending on etiology, BVP can have a rapid or slowly progressive onset (mostly due to ototoxicity and genetic causes respectively). BVP can also develop following recurrent episodes of vertigo, as is particularly seen in patients with bilateral (sequential or consecutive) Menière’s Disease (7).

**Table 1:** Etiologies of bilateral vestibulopathy (18, 19).

Idiopathic	
Genetic	DFNA9, DFNA11, DFNA15, DFNB4, mutation chromosome 5q, 6q, 11q, 22q Muckle Wells (NLPR3)
Toxic/metabolic	Antibiotics (particularly aminoglycosides), furosemide, amiodarone, aspirin, chemotherapeutics (e.g., cisplatin), immunotherapy (e.g., immune checkpoint inhibitors), anti-epileptic drugs (particularly aromatic anti-epileptic drugs), alcohol, styrene poisoning, combination non-steroidal anti-inflammatory drugs with penicillin
Infectious	Meningitis, syphilis, Lyme disease, bilateral vestibular neuritis (Herpes Simplex Virus), Herpes zoster, rubella
Other ear pathology	Bilateral Menière’s disease, otosclerosis, bilateral labyrinthitis, cholesteatoma, vestibular atelectasis, presbyvestibulopathy
Trauma	Head trauma, iatrogenic (e.g., bilateral Cochlear Implant, local radiotherapy)
Autoimmune	Cogan’s syndome, Susac syndrome, Sarcoïdosis, Granulomatosis with polyangiitis, Sjögren syndrome, inflammatory bowel disease, Behçet’s disease, celiac disease, polyarteritis nodosa, antiphospholipid syndrome, Anti-GQ1b antibody syndrome, Autoimmune Inner Ear Disease, other systemic diseases
Neuropathies	Guillain-Barre Syndrome (GBS), and Chronic Inflammatory Demyelinating Polyradiculoneuropathy (CIDP), Charcot–Marie Tooth (CMT) disease, Fabry’s disease
Neurodegenerative	CANVAS, Friedreich Ataxia, multiple system atrophy, SCA3, SCA6, SCA27B,
Congenital/syndromal	Usher, Turner, enlarged vestibular aqueduct syndrome, Alport syndrome, coloboma-heart-atresia-retarded-genital-ear (CHARGE) syndrome
Vascular	Vertebrobasilar dolichoectasia
Tumors	Bilateral vestibular schwannoma, Neurofibromatosis type 2, metastasis, lymphoma
Other	Auditory neuropathy spectrum disorders, superficial siderosis, hypothyroidism, vitamin B12 deficiency, folate deficiency, vestibular migraine, Wernicke’s encephalopathy.

### 2.2 Symptoms

Two of the main physical symptoms of BVP are movement-induced blurred vision (oscillopsia) and unsteadiness when walking or standing which often worsens on uneven ground or in darkness. These symptoms are primarily due to impaired vestibular-ocular and vestibular-spinal reflexes (6). Furthermore, BVP may be associated with cognitive and emotional symptoms such as difficulties with performing dual tasks, impaired concentration, forgetfulness, reduced spatial orientation, anxiety, anger, and sadness (26–29).

Neither vertigo nor abnormal nystagmus are typical symptoms of BVP as both are generally related to an acute asymmetry in vestibular function (i.e., an acute unilateral vestibulopathy) and are in general not caused by a symmetrical decrease in vestibular function (30). The exception here is bilateral sequential vestibulo-ocular reflex (VOR) reduction. In other words, vertigo and nystagmus can be related to the underlying etiology of BVP (e.g., Menière’s disease), but are generally not a sign of BVP itself.

In particular the unsteadiness can be difficult to recognize as balance control is a multisensory process (31–33). Compensation via sensory reweighting plays a key role in attempted recovery from BVP. In this process, the remaining senses such as vision, somatosensory input (e.g., pressure perception) and proprioception are preferentially utilized (34). As a result of sensory reweighting, many spatiotemporal gait parameters do not differ between BVP patients and healthy controls at their preferred walking speed. However, BVP patients do tend to walk with an increased cadence (35). When testing gait at fixed walking speeds, gait parameters such as step length and step width variability differ significantly to those of healthy controls (33). Sensory reweighting also explains why certain complaints worsen in situations where other sensory inputs are less effective, such as worsening of unsteadiness in poorly lit environments. This phenomenon offers a partial explanation for the higher incidence of falls and severe fall-related injuries in the BVP population (18, 36–38). In addition, loss of somatosensory input (in particular from the soles of the feet) also increases unsteadiness and is a proven risk factor for falls in BVP patients (39). Other risk factors for falls include advanced age, a decline in cognitive resources and having a sedentary lifestyle (38).

Due to the absence of standardized and validated Patient Reported Outcome Measures (PROMs) capable of capturing the subjective severity and burden of the complete spectrum of BVP symptoms, the Bilateral Vestibulopathy Questionnaire (BVQ) was recently developed. The BVQ serves as a comprehensive tool for assessing the spectrum of BVP symptoms and its impact on daily life, in order to quantify treatment efficacy and improve clinical decision making (40, 41).

## 3 Physical examination and laboratory assessment

Physical and laboratory assessment in BVP patients mainly focuses on two aspects: (1) identifying the presence or absence of central vestibular signs (e.g., gaze evoked nystagmus, downbeat nystagmus, dysmetria, etc.), and (2) confirming BVP.

### 3.1 Physical examination

In identifying central vestibular signs, it is advised to perform cerebellar testing, including oculomotor examination, evaluation of coordination (e.g., finger-to-nose test for identifying dysmetria, rapid alternating movements for identifying dysdiadochokinesia) and evaluation of gait and posture. As abnormalities in oculomotor functioning may be the first signs of central pathology, oculomotor examination should always be performed (42). The Head Impulse Test (HIT) is sensitive in identification of severe BVP, particularly when performed by an expert (43). However, false-negative results may be found in the presence of covert saccades, mild BVP and when the HIT is performed by less experienced clinicians (5, 43, 44). Another key oculomotor test is the visually enhanced VOR (VVOR), which is specific for the combination of BVP and cerebellar impairment. The VVOR is performed by turning a patient’s head slowly side-to-side while the patient fixates at an earth-fixed target (e.g., the clinicians nose). The VVOR is abnormal in case the ensuing eye movements are broken-up or saccadic, rather than smooth. The VVOR is a simple, brief and reproducible bedside test (45). In addition to oculomotor examination and the HIT, Romberg’s test (including Romberg in tandem or Romberg on foam rubber) and evaluation for neuropathy is recommended (39, 46, 47).

### 3.2 Laboratory assessment

The Consensus document of the Classification Committee of the Bárány Society describes the diagnostic criteria for BVP as summarized in Table 2 (6). Regarding the three objective VOR test measurements (Table 2, part C), both caloric testing and horizontal vHIT appear to be more sensitive for detecting impairment of vestibular function than the torsion swing test (rotatory chair testing). The latter proved to be most sensitive in measuring residual vestibular function (19). When performing the vHIT, it is important to be aware that the sensitivity may depend on the type of device used, as vHIT systems are not yet standardized across different manufacturers (48). In addition to the HIT, the Suppression Head Impulse Paradigm (SHIMP) was introduced as a diagnostic tool for identifying VOR alterations in BVP patients. The advantage of SHIMP is that it significantly reduces covert saccades (49, 50), which might allow for more reliable VOR gain calculation. However, a recent study in BVP patients showed that the clinical benefit of SHIMP compared to HIT was marginal, given that both paradigms successfully detected BVP in the majority of patients (93%) (50). Despite the comparable diagnostic capabilities of SHIMP and HIT, the former, characterized as a ‘covert saccade killer’, may serve as a viable alternative in clinical settings where access to a vHIT-system is unavailable (50). In order to facilitate the most efficient diagnostic workflow, it is worth considering to first perform vHIT (due to the lower patient burden), followed by caloric testing, before performing the torsion swing test. In this way, the test battery can be discontinued as soon as the patient meets one of the diagnostic test criteria.

**Table 2:** Diagnostic criteria for bilateral vestibulopathy, as described by the Bárány Society (6).

A. Chronic vestibular syndrome with the following symptoms	Unsteadiness when walking or standing plus at least one of 2 or 3
	Movement-induced blurred vision or oscillopsia during walking or quick head/body movements and/or
	Worsening of unsteadiness in darkness and/or on uneven ground
B. No symptoms while sitting or lying down under static conditions	
C. Bilaterally reduced or absent angular VOR function documented by	Bilaterally pathological horizontal angular VOR gain <0.6, measured by the video-HIT or scleral-coil technique and/or
	Reduced caloric response (sum of bithermal max. Peak SPV on each side <6°/sec) and/or
	Reduced horizontal angular VOR gain <0.1 upon sinusoidal stimulation on a rotatory chair (0.1Hz, Vmax = 50°/sec) and a phase lead >68 degrees (time constant <5sec).
D. Not better accounted for by another disease	

Other possible vestibular function measurements are cervical and ocular Vestibular Evoked Myogenic Potentials (c- and oVEMPs). However, various studies have found a high degree of variability in VEMP responses within BVP populations, and more importantly, there remains a lack of certainty regarding whether isolated bilateral impairment of both otolith organs causes significant disability (19, 51, 52). Therefore, c- and oVEMPs are as yet not included in the Bárány diagnostic criteria as a definite stand-alone diagnostic modality in BVP.

Several outcome measures are available for quantifying the functional manifestations of BVP. The functional HIT (fHIT) proved to be a feasible test for evaluating oscillopsia by testing the Dynamic Visual Acuity (DVA) (53). Another assessment complementary to the fHIT, is testing the DVA while walking on a treadmill, which is strongly related to activities of daily living and therefore has significant ecological validity (54, 55). Unfortunately, the DVA while walking on a treadmill cannot always be performed in elderly patients, as increased age in combination with BVP leads to a higher drop out rate during test performance (54). Lastly, the vestibular system contributes to detecting self-motion. Earlier research showed that self-motion perception is significantly decreased in patients with BVP compared to control subjects, and therefore self-motion perception could also be considered as a functional outcome measure in the future (56–58).

## 4 Proposal of a diagnostic algorithm for BVP

**Figure 1:**
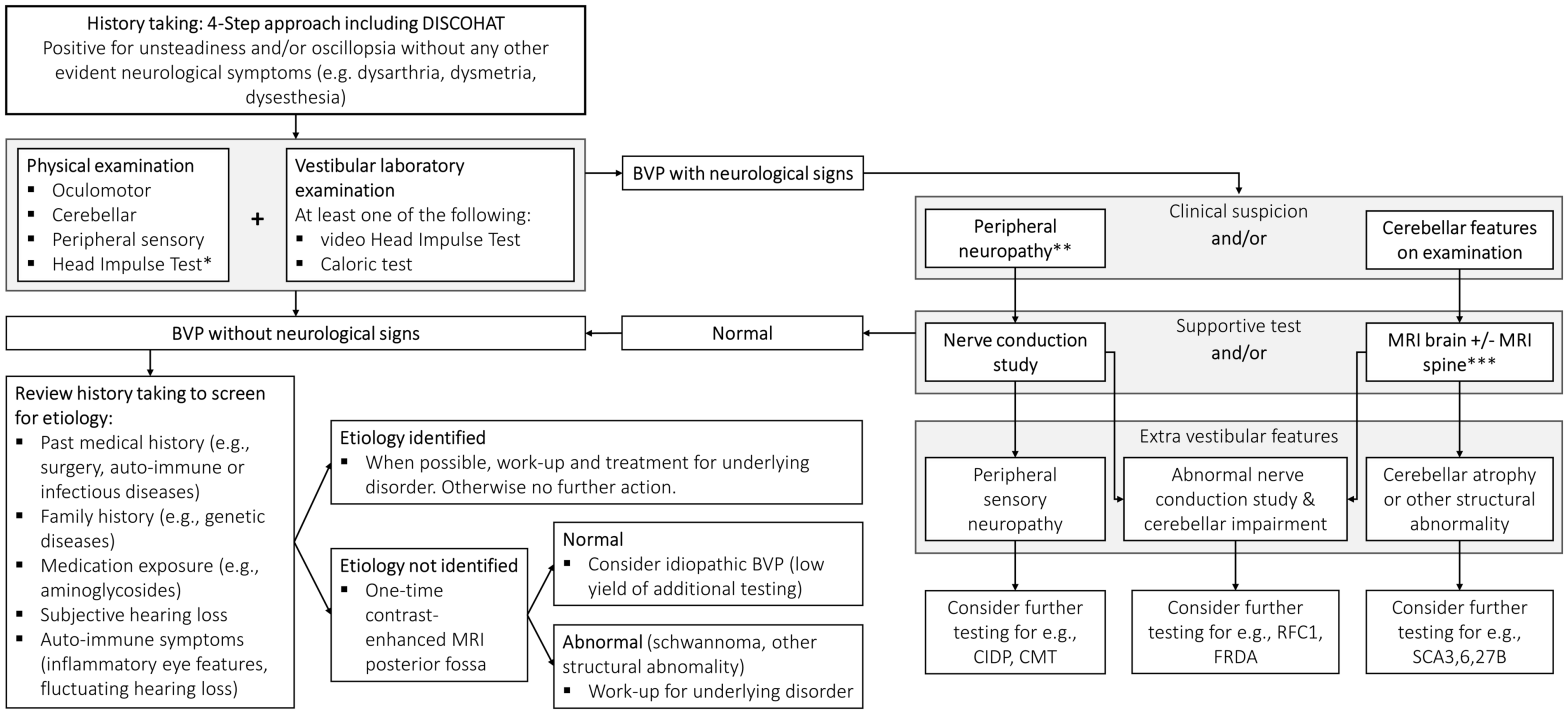
A new diagnostic protocol for bilateral vestibulopathy (BVP). The 4-step approach is discussed elaborately elsewhere (59). DISCOHAT = the acronym of ‘Darkness worsens symptoms, Imbalance, Supermarket effect, Cognitive complaints, Oscillopsia, Head movements worsen symptoms, Autonomic complaints, Tiredness’. *Be aware that the presence of covert saccades, mild BVP and HIT by nonexperts can result in false-negative results. **Suspected based on sensory examination findings and/or imbalance out of proportion to isolated BVP. ***MRI abnormalities are not required as clinical signs, e.g., oculomotor abnormalities, may precede MRI cerebellar changes.

Establishing the diagnosis of BVP is often delayed. To facilitate a prompt, accurate and robust diagnostic process, a new protocol has been proposed based on the current knowledge summarized in this narrative review (Figure 1). The diagnostic process for vestibular disorders starts with an adequate medical history. A tool for improving history taking is the 4-step approach which focuses on: (1) potential attacks of vertigo and/or dizziness, (2) potential chronic vestibular symptoms, (3) any additional functional, psychological or psychiatric co-morbidities, and taken together leading to (4) a comprehensive differential diagnosis (59). Regarding episodes of vertigo or dizziness attacks in the context of BVP principally depends on etiology (e.g., positive history taking for experiencing vertigo attacks in a patient with bilateral Menière’s Disease). Chronic symptoms are however always present in BVP and can be summarized according to the DISCOHAT acronym (worsening of symptoms in Darkness and/or uneven ground, Imbalance, Supermarket effect, Cognitive complaints, Oscillopsia, Head movements worsen symptoms, Autonomic complaints, and Tiredness), with a particular focus on imbalance/unsteadiness and oscillopsia (60).

In every patient with a positive history for imbalance/unsteadiness and/or oscillopsia without any other neurological symptoms (e.g., dysarthria, dysmetria, dysesthesia), a thorough physical examination focused on oculomotor testing, HIT, cerebellar testing, and testing for neuropathy is indicated. This is all necessary in order to identify patients with combined peripheral and central neurological disorders such as CANVAS or the recently described Spinocerebellar Ataxia Type 27B (SCA27B) (7, 61). Oculomotor abnormalities such as broken-up visual pursuit, gaze-evoked nystagmus and abnormal saccades to target, point to pathology of the cerebellum and its connections (62). In addition to oculomotor signs, other localizing abnormalities observed during examination include cerebellar dysarthria, often described as ‘slurred’ or ‘drunken’ speech, as well as limb ataxia, such as the presence of an intention tremor during the finger-to-nose test. These clinical manifestations are frequently encountered in cases of cerebellar impairment (63).

In addition to the physical examination, at least one vestibular laboratory examination to objectify the vestibular function must be performed, preferably by means of a vHIT or caloric test (5). vHIT is favored over HIT as it provides a calculated VOR gain and recognizes the influence of covert saccades and other eye movement abnormalities (5). Regarding caloric testing, it is important to irrigate with at least 250 mL of water for a duration of 30s for both cold (30°C) and warm (44°C) irrigations with a 5-min stimulus interval between irrigations. Furthermore, it is necessary to not only look for a potential asymmetry (%) but also evaluate absolute caloric values (°/sec). The torsion swing test is less suitable to use as a single diagnostic tool since it appears to be less sensitive for detecting vestibular impairment as compared to vHIT and the caloric test (7, 19). Therefore, the torsion swing test is not included in the diagnostic algorithm.

Where history, physical examination and vestibular function tests lead to a BVP diagnosis without neurological involvement, the next step is attempting to identify the etiology (Figure 1, lower left side of the flow chart). Important information includes past medical history (e.g., surgery, auto-immunity, infectious diseases such as Lyme disease or syphilis), family history (genetic disorders), use of medication (ototoxicity), subjective hearing loss and auto-immune symptoms (including those of inflammatory eye disease and fluctuating hearing loss). Where a treatable etiology is identified (e.g., autoimmune or infectious disease), then this obviously becomes the clinical priority. Where the etiology remains idiopathic, a one-time contrast-enhanced MRI of the posterior fossa is advised because of the relatively high yield of positive findings (e.g., vestibular schwannoma). Contrast-enhanced MRI scans are preferred over non-contrast MRI scans as they increase the detection rate of small schwannomas, particularly intralabyrinthine ones, which may be missed by radiologists who are less familiar with intralabyrinthine pathology (64). Blood tests are not routinely advised because of the low yield (7).

Where history, physical examination, and vestibular function tests lead to a BVP diagnosis in combination with neurological signs, the next step is the evaluation of peripheral sensory neuropathy and/or cerebellar features by performing clinical tests such as a nerve conduction study and/or a MRI scan (Figure 1, right side of the flow chart).

Regarding peripheral neuropathy, studies show that up to 53 percent of patients with a peripheral neuropathy also suffer from vestibular hypofunction (65). Conditions where this combination is seen, include Chronic Inflammatory Demyelinating Polyradiculoneuropathy (CIDP), Charcot–Marie Tooth (CMT) disease, Guillain-Barre Syndrome (GBS), neurosarcoidosis and other inflammatory and inherited diseases (noting that diseases such as GBS, CIDP and neurosarcoidosis require treatment which may be lifesaving) (66–71). Therefore, further testing for, e.g., CIDP and CMT needs to be considered in cases of bilateral vestibulopathy accompanied by abnormal nerve conduction studies indicating a peripheral sensory neuropathy.

Regarding cerebellar features, BVP is increasingly identified as an extracerebellar feature of the many cerebellar ataxias, including the most common sporadic and inherited diseases such as idiopathic late-onset cerebellar ataxia (ILOCA), idiopathic Cerebellar Ataxia with Bilateral Vestibulopathy (iCABV), spinocerebellar ataxia (SCA) 3 and 6, Friedreich ataxia (FRDA), Cerebellar Ataxia, Neuronopathy, Vestibular Areflexia syndrome (CANVAS)/*RFC1*-related disease, and most recently SCA27B (*FGF14* GAA expansion) (23, 61, 72–76). Where cerebellar signs on examination, or MRI changes such as atrophy are found (with or without sensory peripheral neuropathy), further testing for the above-mentioned etiologies is advised. It is important to bear in mind that cerebellar signs on examination (particularly oculomotor abnormalities) may be seen well before MRI changes are found. In other words, the normal appearance of the cerebellum on MRI scanning does not exclude cerebellar impairment, especially in the earlier stages of cerebellar disease (77).

## 5 Treatment

Unfortunately, to date, the prognosis for recovery of vestibular function is poor (14). Detailed patient counseling and education with a focus on explaining the cause of the symptoms is therefore of great importance.

Vestibular rehabilitation therapy remains the mainstay of treatment for vestibular hypofunction. Exercise-based vestibular rehabilitation is aimed at (1) adaptation and (2) substitution. Adaptation is the process by which the gain of the vestibular reflexes are increased, while substitution (or sensory reweighting) involves strategies to utilize alternate modalities in place of the vestibular hypofunction (78). The reported efficacy of vestibular rehabilitation in BVP differs. Two independent systematic reviews found moderate to strong evidence supporting the utility of vestibular rehabilitation in BVP in improving gaze and postural stability and improving overall functional status (79, 80). Additionally, vestibular rehabilitation was found to significantly reduce the number of falls in patients with combined BVP and cerebellar impairment (81). Sensory reweighting (substitution) is however limited since other somatosensory systems cannot fully compensate for the elaborate function of the vestibular system. In particular, the somatosensory system is not able to respond as rapidly as the VOR, the vestibulo-spinal reflex, and the vestibulo-collic reflex. As a result, the balance system as a whole lacks the speed and automatism provided by an intact vestibular system (82). In other words, a BVP patient is less able to reflexively react to balance perturbations. Sensory substitution devices aim to substitute the loss of vestibular input by administering tactile or auditory stimulation which may result in some degree of improvement in balance control (83–85). However, it is important to note that these devices are unable to replace the rapid vestibular reflexes.

Other therapeutic approaches, such as noisy galvanic vestibular stimulation, aim to enhance the residual vestibular function. Previous studies indicated that noisy galvanic vestibular stimulation improves postural and gait stability in patients with BVP (86–88). This treatment strategy will probably offer the most benefit in patients with residual vestibular function (e.g., similar to the functionality of hearing aids: hearing aids can augment the hearing performance only in the presence of residual hearing).

An artificial balance organ, the vestibular implant, directly stimulates the peripheral vestibular nerve and therefore does not depend on the presence of residual vestibular function (89, 90). Vestibular implant research to date demonstrated partial recovery of the VOR and the vestibulo-collic reflex, and hence, rapid vestibular responses are achievable (89, 91–93). This approach appears promising since the functional improvements closely match the expectations of BVP patients regarding vestibular implant treatment (94, 95).

## 6 Conclusion

The knowledge of BVP has grown expansively since its first description in 1936. The proposed diagnostic algorithm facilitates in-clinic assessment and diagnosis. In addition to the vestibular rehabilitation, therapeutic modalities currently under development hold significant promise.

## Author contributions

LS: Conceptualization, Methodology, Writing – original draft, Writing – review & editing. DS: Writing – review & editing. NG: Writing – review & editing. AF: Writing – review & editing. VR: Writing – review & editing. RB: Conceptualization, Methodology, Supervision, Writing – review & editing.
